# Supplementary material for: Are carbon emissions trading and green financial instruments synergistic? -Comprehensive quantitative research based on content analysis
Source: PLoS One. 2024 Mar 7;19(3):e0298601. doi: 10.1371/journal.pone.0298601 (PMC10919601; doi:10.1371/journal.pone.0298601)
Supplement: S1 Data — (ZIP) [file pone.0298601.s001.zip › Data/地级市Z_F工作报告.zip!/╣π╢1⁄2/╢1⁄2▌╕╩╨/└┤╫╘╣1⁄2╓┌║┼í╛┬φ┐╦╩2╛▌═°í┐_2004.docx]

各位代表：

现在，我代表市人民政府，向大会作工作报告，请予审议，并请政协各位委员和其他列席人员提出意见。

2004年工作回顾

过去的一年，我们认真贯彻邓小平理论和“三个代表”重要思想，树立和落实科学发展观，坚持开拓创新、团结拼搏，较好应对了供地困难、水电不足、用工短缺等问题，圆满完成了市十三届人大一次会议确定的各项任务。

————发展观念逐步转变。党的十六届三中全会提出了科学发展观，市第十一次党代会提出了“一城三创五争先”的工作新思路。我们认真学习贯彻，逐步树立起科学发展观和正确的政绩观，主动适应中央加强宏观调控的新形势，积极创新发展模式、创新发展环境、创新发展能力，促进了全市经济社会和人的全面发展。

————综合实力持续增强。全市生产总值1155.3亿元，比上年增长19.6%；固定资产投资427.8亿元，增长34%；社会消费品零售总额389亿元，增长15.1%；进出口总额645.2亿美元，增长24%，其中出口351.9亿美元，增长26.1%；财政总收入259.1亿元，增长25.5%，其中市地方财政收入82.6亿元，增长22.5%；年末本外币各项存款余额2576.9亿元，比年初增长15.2%，各项贷款余额1450亿元，增长16.3%。东莞综合实力跃居中国百强城市第十二位、地级市第一位。

————城市品位显著提升。荣获“中国综合实力百强城市”、“中国最佳魅力城市”、“中国最具经济活力城市”、“中国优秀旅游城市”、“全国双拥模范城”、“全国科技进步先进市”、“全国无偿献血先进城市”、“国家电子信息产业基地” 、“国家篮球城市”等称号，还通过了创建“国家卫生城市”的国家考核。东莞在国内外的知名度迅速提高，制造业名城、生态绿城和文化新城的形象深入人心。

————宏观调控取得成效。经济社会在调整中发展，在发展中提高。固定资产投资增速比上年回落32.7个百分点，金融机构贷款余额增速回落12.8个百分点。居民消费价格总水平涨幅控制在3.0%。三大产业比例由上年的3.0∶54.0∶43.0，调整为2.4∶55.4∶42.2。全年城镇居民人均可支配收入20526元，增长11.1%；农村居民人均纯收入9083元，增长7.1%。

总结我们的工作，主要是抓好“七个统筹”：

一是统筹城乡发展，加快城市化进程

加快城市建设步伐。编制完成中心城区公共停车场等多项规划，审批通过多项镇（区）总体规划和详细规划。建成市行政办事中心、会议大厦、高级中学、图书馆等12项重点工程，组织民间资金建成第一国际、会展酒店等项目，完善了城市新区功能。改造市域四条主干道和市区一批马路，建成松山湖大道、环城路等，新增公路通车里程71公里。投资7.67亿元新建改建供水工程。评出东莞新八景和文物八景，重建修缮部分景点，改善了市容市貌。

加大城市管理和经营力度。实施城市管理强化工程，重点整治卫生、交通等问题。建立市区、镇和跨镇三级公交服务体系，市区城巴线路覆盖环城路以内95%的主干道；26个镇实施镇内公交发展规划，已投放运力771辆。完善土地收购储备工作，全年收购、征用土地333亩，储存总量1.4万多亩。完善有形土地市场交易制度，一级、二级市场共出让、转让6235亩，总成交价30亿元。公开出让17个石场的开采权，总成交价1亿元。公开出让大型立柱广告经营点使用权，收入1087万元。

加快推进农村城市化。重点在8个中心镇开展“村改居”，累计完成100个村的改制，占当年符合改制条件村总数的56.8%。稳步推进农村股份合作制改革，155个村、726个组完成改制，分别占全市总数的27.7%和29.3%。积极推进植树绿化、环境净化和旧村改造工作，全年改造旧村16个，完成拆迁面积3.6万平方米，在建农民公寓村14个，建筑面积126万平方米，入住农户1110户。

二是统筹经济增长的速度质量与效益，优化产业结构

推进农业产业化。培育壮大农业龙头企业，新增国家重点农业龙头企业1家、市级2家。全市龙头企业总产值7亿元，带动农户1.8万户。实行粮食工作责任制考评，落实农民种粮直补政策。全年粮食播种面积8.1万亩，总产3万吨。全市农业总产值44.2亿元，比上年增长1.8%。

坚持以信息化带动工业化，以工业化促进信息化。制订经济社会信息化规划，加快建设信息化基础设施，完善全市电子政务平台，建成东莞企业信用网。优先发展以电子信息产业为主导的高新技术产业，大力实施制造业信息化、绿色化和标准化工程，积极应用高新技术和先进适用技术改造提升传统产业，培育印刷、玩具等特色产业，淘汰落后产业。新增省级高新技术企业53家，累计201家；新认定省级民营科技企业70家、市级200家。投入技术改造资金41亿元，重点支持大中型企业、装备制造企业以及电力企业的技术改造。产业发展的质量和效益得到提升。

提高三产发展水平。大力发展现代物流业，抓紧编制商业网点规划。引导会展业健康发展，举办各类展会66场，交易总额350亿元。整合推介旅游资源，改善旅游设施，提高旅游接待能力和服务水平。旅行社增至26家，星级酒店增至85家，其中五星级13家。全年接待游客1131万人次，旅游总收入85.7亿元。深化地方金融机构改革，引进首家外资金融机构星展银行东莞代表处，发展利用资本市场，防范化解金融风险， 全市金融机构不良贷款率降到7%以下。

三是统筹对内发展和对外开放，推进内外源型经济协调发展

掀起民营经济发展新高潮。落实培育发展“两自”企业的政策措施，引导民营企业走“品牌强企”和“科技强企”之路。全市民营企业新创国家级、省级名牌产品和驰（著）名商标41个。向民间资本开放一批环保、交通、能源、文化等项目，建立健全民营融资辅导中心等机构，协助组建民营企业集团30家，累计60家。积极引导民营企业“走出去”，新增民营进出口企业407家、民营境外投资企业2家。年末全市注册个体户和私营企业24.7万户，增长14.2%，注册资金447.9亿元，增长27%；全年上缴各项工商税收45.2亿元，增长34.7%，占全市的19.8%。全市有17家企业入选2004年“中国民营企业进出口百强”，并包揽前三名，3家企业入选“广东省百强民营企业”。

开创外源型经济发展新局面。创新招商方式，主动联合香港等地有关机构，赴国外推介招商。提高引资门槛，严禁擅自建设无项目标准厂房，有效提升了我市利用外资的质量和水平。全年新签外商投资项目1423宗，比上年减少101宗，但合同利用外资41.3亿美元，增长26.9%；实际利用外资30.3亿美元，增长18.4%。新签投资额超千万美元的项目45宗，增加12宗。新增世界500强企业的投资项目4宗。推行加工贸易电子联网监管，完善集中服务日、关贸协调会议等制度，建立与世界500强企业合作协调工作机制，促进了现有企业增资扩产。全年增资扩产项目8487宗，增资合同金额25.9亿美元，分别增长1.8倍 和56.5%。扩大引资领域，引进了港澳服务业投资项目19宗，引进了深圳、上海、天津等市企业投资项目一批。全面落实出口退税改革政策，促进了对外经贸发展。

四是统筹区域发展，扶持龙头和中心以及欠发达地区

打造三大龙头。认真贯彻中央宏观调控政策，整合、撤并、压缩全市工业园区，重点开发建设松山湖科技产业园、虎门港和东部工业园。松山湖科技产业园建成100多公里道路沿线的水电、通讯、绿化等工程，建成100多万平方米的公共配套建筑，引进上规模、高档次项目32宗，引进留学生企业和科研机构30家，协议引资10多亿美元。虎门港进一步理顺了各方关系，首个深水码头获得国家核准，引进投资项目8宗，总投资31.2亿元，全年港口货物吞吐量2600万吨，增长10.5%，出入境旅客40多万人次，增长98.2%，征收关税12亿元。东部工业园也积极整合报批。三大龙头的顺利开发，带动了周边地区发展，较好地实现了全市东中西三线联动的战略设想。

扶持欠发达地区加快发展。扶贫工业基地建设方面，发放扶贫贷款9.1亿元，支付贷款贴息6959.2万元，返还用地收费3685.6万元，帮助11个欠发达镇新建厂房81.2万平方米，新增企业190家、投资总额75亿元，为镇级财政增收1.8亿元。基础设施建设方面，发放2840.9万元，支持欠发达村和革命老区改善基本生产生活条件；发放2085万元，支持26个欠发达村开展联合办学。债务化解方面，通过闲置土地处理、债务重组等方式，帮助镇村化解债务4.6亿元，81个欠发达村资产负债率52.2%，下降7.3个百分点，26个欠发达村实现脱贫。结对帮扶方面，帮扶单位共援助欠发达镇村资金2045.3万元，7980名干部参与“千干扶千户”活动，捐助资金641.6万元，解决实际困难4679宗。欠发达地区利用外资和工商税收等多项经济指标的增速，超过先发展地区。

支持中心城镇发展。及时减免和返还各中心镇税费；督促加快编制总体规划，已有7个中心镇的总体规划通过市政府审批；加快建设常虎高速、东部快速和西部干道，支持常平镇改造东莞东火车站广场，增强了中心镇的聚集和辐射功能。

五是统筹人与自然和谐发展，建设生态绿城

全面铺开整山工程。按照整体规划、分区建管、保护生态、分期开发、路网先行的原则，建设黄旗山、水濂山、大岭山、大屏嶂、同沙等五个森林公园。全年完成投资3.6亿元，建成车行道路52公里，步行道路39公里，出入口和广场18个，景点17个。

加快建设环保基础设施。编制完成环保规划8项；投资7.9亿元，建成市区污水处理厂二期、樟村水质净化厂等污水处理工程9项，新增日处理能力287万吨；扩建了厚街垃圾焚烧发电厂，完成了市区、横沥两座垃圾焚烧发电厂的土建工程和16家污水处理厂的BOT招标。全市污水处理率提高到12.4%，垃圾无害化处理率提高到9.5%。

加强环保综合整治。清理养殖场3812个、生猪26.8万头，整治内河涌61条（段）共195公里，清拆窝棚窝点3535个共41.4万平方米，复绿采石场90个共9.8万平方米，关闭和拆除水泥厂34家。拒批选址不宜、污染严重的项目431个，实行“三同时”验收项目620个，征收排污费6789万元，查处各类环境违法案件460宗。建成石马河调污工程，有效保护东江水质。扎实推进城乡水利防灾减灾工程建设，被省评为先进。

六是统筹改革发展与稳定，构建和谐东莞

坚持以改革促发展。深化企业改革，完成一般竞争性领域市属企业转制39户，支持市属资本全资控股企业做大做强。撤并9家市属资产经营有限公司为1家，着力解决市属关停企业债务等问题。深化农村税费改革，在全省率先免征农业税，实现农民税费零负担。深化财税体制改革，实施全口径预算管理，国库集中收付制度推广到所有政府组成部门及其下属单位。审核财政投资项目159宗，核减不合理支出6.3亿元。深化政府机构改革，理顺职能关系，精简审批事项。改革公务用车制度，拍卖市直单位原有车辆，发放公务交通补贴。清理评比达标表彰项目199个，撤销90个。完成30个单位（项目）的审计监督。推进投融资体制改 革，加强投资管理，遏制盲目投资。深化价格和收费改革，取消行政事业性收费12项，减轻了企业和群众负担。

坚持以稳定保发展。加强公安队伍建设，新增公安编制2700名。狠抓社会治安综合治理，开展打击街头犯罪等多次专项行动，全年侦破刑事案件数比上年增长20.1%，破案率提高3.8%。改革出租屋及租住人员管理模式。落实人口与计划生育工作责任制，全面完成省下达的任务，户籍人口出生率10.86‰，自然增长率5.96‰，计划生育率93.48%。高度重视信访工作，建立处理信访突出问题及群体性事件联席会议制度和信访督查专员制度。落实安全生产责任制，加强宣传和培训，提高注册安全主任和特种工持证上岗率，全年检查和整改各类事故隐患3000多宗，有效遏制了重特大安全事故的发生，全市安全生产主要指标全面下降。继续整顿规范市场经济秩序。加强食品安全管理，初步建成农产品质量检测体系。坚决打击走私贩私，全年查处案件348宗，案值23.2亿元。清理兑付拖欠农民征地补偿款1.45亿元。清还拖欠和克 扣的农民工工资9863万元。清退办理暂住证过程中的乱收费157万元。查处公路“三乱”问题18宗。积极推进征兵、人防、民兵预备役、国防动员教育工作，扎实开展双拥共建。

坚持在发展中求稳定。出台改制企业职工安置配套新政策，保障了企业五类人员、退休干部及其他弱势群体的利益。建立全市统筹的农（居）民基本医疗保险制度。年末全市各项保险参保人数达643.8万人次，比年初增长21.2%。提高城乡居民最低生活保障标准，发放低保金3652万元。帮助630户困难群众修建、改造住房2.8万平方米。市镇公共就业服务机构免费推荐就业2.6万人次，提供免费和资助性就业技能培训8876人次，帮助1.7万多人成功就业。年末城镇登记失业率为1.7%。

七是统筹经济社会发展，打造文化新城

加快发展文化事业和产业。创新文化发展观念和工作思路，实施文化与群众、文化与经济、文化与城市的零距离行动。创新文化发展机制，实行管文化与办文化分离。在全国率先举办公益文化项目招商，签约项目73个，金额4360多万元。制订市属六大文化设施筹建运作方案，建立基层文化建设考评制度，促使基层文化建设取得突破性进展，全市有24个镇（区）文化站被评为省特级文化站，比上年增加了1倍。全市举办各类文艺晚会478场、专项文艺比赛225个、文化展览288期，参与人数200多万人次。全市138个文化广场举办广场舞活动1.4万场次，广场推介活动122次，参与人数1000多万人次。创新鼓励扶持文艺创作的政策措施，颁发了首届东莞文学艺术玉兰大奖，设立五大文艺创作基地，本市文艺作品赢得25个国家级奖项和61个省级奖项。加强文物资源的挖掘、保护和整合利用，公布不可移动文物79处及其保护范围和建设控制地带，编印《东莞历代书画选》。加强文化市场管理，全年受理群众投诉举报460宗，出动稽查625次，办结案件392宗。创新舆论宣传方式，开设东莞论坛，开通东莞有线数字电视和东莞电台阳光网站，重点加强了公务员和未成年人等群体的思想道德建设。新闻出版、档案方志等工作也取得新的成绩。

大力实施科教兴市和人才强市战略。组建市科技投资担保公司，为企业解决发展资金2.3亿元，其中科技企业占65%。举办东莞国际科技合作周，组织民营科技企业深入著名高校洽谈，推动了产学研合作。推进创新平台建设，新增省级企业工程技术研究开发中心3个、市级10个、省级企业技术中心2个、专业镇技术创新平台和信息化平台各1个。优化市属学校布局，扩大东莞中学等名校规模。普通高考录取率96.9%，创历史新高，连续两年居全省地级以上市第一位。创建省教育强镇4个。建立人才资本产权激励制度，成立镇（区）人才服务站，增设人事代理点，改善了引智环境。举办第二届东莞发展与百名博士创业洽谈会，建立我市首个企业博士后工作站，加快了引智步伐。全年引进各类人才11.9万人，全市人才总量达到71.2万人。

积极发展卫生体育等社会事业。制订公共卫生体系建设规划，市、镇（区）和医院三方投资，加快建设相应机制、队伍和设施。年末全市持证医疗机构1074家，医疗卫生技术人员2.1万人，床位1.1万张。依法整顿和规范医疗市场，查处和取缔非法行医窝点2241个，清理无证行医人员665人，没收非法行医药品药械价值211.5万元。成功预防高致病性禽流感。广泛开展群众性体育活动，努力提高竞技体育水平，全年举办市级运动会9次，镇（区）、部门级运动会10次。莞籍运动员夺得国际赛金牌49枚、亚洲赛金牌1枚、全国赛金牌34枚、省赛金牌93枚。积极发展体育产业，现有注册体育健身企业500多家，经营项目27个；体育彩票销售收入1.5亿元。积极参与泛珠三角区域合作，与广西北海市结为友好城市。积极开展对口帮扶，援助河池、韶关和新疆、西藏等有关地区财物6765万元，援建学校27所，组织经贸合作项目一批。此外，还顺利推进了外事侨务、民族宗教、工青妇幼、台务、统计、残疾、老龄等工作。

各位代表！过去一年取得的成绩，是上级党委和政府以及东莞市委正确领导的结果，是全市人民、广大外来员工和驻莞部队齐心实干的结果，也与市人大、市政协的监督支持分不开，与香港和澳门特区同胞、台湾同胞、海外侨胞以及国际友人的关心帮助分不开。在此，我代表市政府，向所有参与、支持和关心东莞现代化建设的各界人士，表示衷心的感谢！

在看到成绩的同时，我们也清醒地认识到，我市经济社会发展中仍然存在一些问题，主要是：城市化滞后于工业化、社会发展滞后于经济发展、城市管理滞后于城市建设、人的素质提高滞后于经济社会发展。具体表现为：高消耗、高污染、低效益的粗放型经济增长方式没有根本性转变；资源供求矛盾比较突出，供地、供电、供水、用工不足；经济社会管理任务繁重，尤其是治安、交通、劳动和环保等方面压力很大，等等。对于这些问题，我们将采取更加有力、更加有效的措施，加快解决。

2005年工作安排

今年，是巩固和发展宏观调控成果、保持经济社会发展良好态势、实现“五年见新城”目标、加强基层基础建设的关键一年。做好今年工作，对于全面实现“十五”计划目标，为“十一五”经济社会发展奠定坚实基础，具有十分重要的意义。

今年市政府工作的指导思想是：高举邓小平理论和“三个代表”重要思想伟大旗帜，认真贯彻党的十六大和十六届三中、四中全会以及省委九届六次全会精神，全面落实市委十一届二次全会的工作部署，坚持树立和落实科学发展观，坚持加强和改善宏观调控，坚持“抓落实、重提高、促发展”的方针，坚持“一城三创五争先”的工作思路，全面推进社会主义物质文明、政治文明和精神文明建设，确保经济社会全面协调可持续发展。

今年全市经济社会发展的主要预期目标是：生产总值增长15%，地方财政收入增长15%，工业总产值增长18%，固定资产投资增长20%，社会消费品零售总额增长12%，实际利用外资增长12%，外贸出口增长12%，人口自然增长率控制在6.8‰以下，城镇居民人均可支配收入增长8%，农村居民人均纯收入增长5%，居民消费价格总水平涨幅控制在3.8%以内。

抓好今年的工作，必须做好“四篇文章”。一是实施三个创新：继续创新发展模式、创新发展环境、创新发展能力。二是抓住三个关键：经济发展关键在科技，社会发展关键在管理，人的发展关键在教育。三是突出三个重点：巩固农村基层，深化社会管理，构建和谐东莞。四是推进三个转变：转变发展观念，转变政府职能，转变工作作风。

要实现上述目标任务，必须着重抓好“八个提高”：

一、转变经济增长方式，提高经济质量和效益

从粗放型向集约型转变。加强和改善宏观调控，实施稳健的财政政策和稳健的货币政策。按照区别对待、有保有压的原则，把好土地审批和信贷投放两道闸门，控制固定资产投资规模。按照“盘活存量、控制增量、提高质量”的原则，大力推进土地集约利用。依法处理闲置土地，盘活用好存量土地。凡市参与投资建设的道路和轨道交通沿线两侧150米范围内的闲置土地，原则上由市土地储备中心依法收购或收回。实行镇（区）领导班子任期建设用地总额控制。制定用地定额管理标准，实行用地投资强度控制。积极为符合供地条件的建设项目尤其是省市重点项目、影响较大急需上马的薄弱环节项目、高科技高附加值产业项目提供用地。

从直线、高耗型向循环、节约型转变。广泛宣传循环经济的理念，大力实施省市联动“绿色制造、清洁生产”科技示范工程，以化工、漂染、电镀、造纸、家具行业以及工业固体废弃物综合处理利用企业为重点，推广“三废”综合利用、再生资源回收利用和可再生能源利用，培植一批清洁生产示范企业。严格限制高能耗、高物耗、高污染的产业，强制淘汰相关设备和产品。大力倡导节约能源资源的生产方式和消费方式，加快建设节约型社会。建立节水节能制度，研究实行梯级水价电价，推广应用节水节电器具。

从劳动密集型向资金技术密集型转变。严守引资最低限额和禁止擅自建设无项目标准厂房两个门槛，从“招商引资”向“招商选资”转变。加快构建区域创新体系，促进科技进步，提高经济效益。以松山湖科技产业园为依托，集聚国家、省和市科技资源，建设一批综合性服务平台，争取在软件、集成电路和生物医药等领域实现重点突破，加快培育高新技术产业。以东莞理工学院等高校为依托，在通信、微电子、新材料、生物医药等领域新建或扩建一批实验室，推进产学研合作。以专业镇为依托，重点建设家具、服装、物流等一批行业性技术创新服务平台，积极应用高新技术和先进适用技术改造提升传统产业。以科技企业尤其是“两自”企业为依托，组建一批企业工程中心和博士后工作站，提升企业研发能力。

二、加快调整经济结构，提高综合竞争力

推动内外源型经济发展协调化。一是推进大开放。实行重点跟踪制度，引导世界500强和国内外其他大企业到我市建立区域总部或投资性公司。用好国家和省市扶持政策，用好广交会平台，鼓励优势企业和产品率先“走出去”。优化服务，引导鼓励符合产业政策的项目增资扩产。不断完善通关设施，增加虎门港一类口岸作业码头，加快建设寮步车检场，组织太平客运口岸搬迁选址论证，争取开通东莞至香港直通货运列车。积极开展加工贸易联网监管系统的开发和试点，对所有加工贸易企业合同实行电子审批，争取对年出口超千万美元的加工贸易企业实施外经贸和海关联网监管。加快转变外贸增长方式，着力推动外贸从注重量的增长向以质取胜转变。积极优化出口商品结构，实施科技兴贸、名牌带动战略，支持有自主知识产权和自有品牌的产品出口，扩大高新技术产品、机电产品、农产品和服务产品的出口，提高传统出口商品的质量和附加值。妥善处理国际贸易摩擦和纠纷。二是推进大融合。把引进外资与提升我市产业结构和技术水平更好地结合起来，引导外资企业扩大本土原材料采购和产品内销业务，引导民营企业提升对外资企业生产经营的配套参与水平，积极开展对引进技术的消化吸收和创新。认真贯彻《国务院关于鼓励支持和引导个体私营等非公有经济发展的若干意见》，继续完善和落实促进民营经济发展的政策措施，提升民营经济整体素质和竞争力。三是推进大合作。积极参与泛珠三角区域合作，拓展发展空间。探索建设产业转移园区。积极参与西部大开发和东北地区等老工业基地的振兴。继续做好市外帮扶工作。

推动产业高级化和适度重型化。提升工业发展层次，重点扶持电子信息、电气机械、纺织服装、家具、玩具、造纸、食品饮料、化工制品等8大支柱产业，提高产业聚集程度，培育一批制造业特色名镇、名企业、名产品、名企业家和名技术专家。优先扶持50家装备制造业重点企业，努力在发展电气机械制造、汽车零配件、精密装备制造和精细化工等方面实现突破。加快发展以光电子、生物医药、新材料等为重点的高新技术产业，争取新认定省级高新技术企业50家、省级民科企业60家、市级民科企业200家。发展大商业，把商业作为未来城市竞争的着力点，加强规划，完善网络，提高档次。搭建大机构，引进跨国公司的采购中心、维修中心、研发中心和营销中心，大力发展以金融、物流、会展、旅游、信息等为重点的现代服务业。改善消费环境，扩大消费需求。认真落实中央一号文件精神，推进农业区域化布局、专业化生产和产业化经营。规划建设麻涌、中堂、桥头等农业生态园区示范点。争取再培育1—2家省级农业龙头企业。继续保护和提高粮食综合生产能力，确保粮食等重要物资的供需衔接。

推动企业规模化和协作化。坚持扶大引强，支持民营企业走优势互补、强强联合的道路，支持组建民营企业集团，引进和培育高科技、高效益、高附加值、高关联度的大项目。坚持扶优汰劣，不断增强中小企业的群体配套能力。用足、用好我市中小企业发展专项资金，推动中小企业技术改造和创新，加快服务体系和信用体系建设，提高中小企业的发展能力。通过广泛的专业化分工和社会化协作，延长和扩展产业链。

三、协调城乡发展，提高城市化水平

加强城市规划。成立城市规划委员会，建立和完善控制性详细规划批前公众咨询和批后公示制度；凡未编制控制性详细规划的地块，一律不予办理规划和用地等手续。继续抓好全市性规划和各镇（区）总体规划的编制工作。

加速构筑“三位一体”大市区。抓紧建设重点工程，继续完善城市中心区功能。续建群众艺术馆等10项工程，新建绿色世界公园等18项工程。做好市区、同沙和松山湖三大组团的连接。优化全市资源配置，促进资源向大市区集中。推进成熟社区建设工程，加强社区服务。四个街道办事处各建一个多功能社区服务中心，辖区至少50%的社区各建一个50平方米以上的社区服务站。四个街道办事处及其管辖的社区基本建立社区管理信息系统。

加快完善基础设施网络。供电方面，组织通明、东城等9台发电机组投入运行，新增装机容量152万千瓦；投资30.8亿元建设电网，新增主变容量1097万千伏安、送电线路329千米；加强电力需求侧管理与电源调度。供水方面，修订城市供水规划，抓紧进行东江筑坝与上移取水口两个方案的对比研究，制订并实施东江与水库联网供水方案，建设第二水厂扩建、第四水厂二期和第六水厂一期等工程，争取日供水量新增105万吨；加快建设厚街水道拦污挡潮工程。交通方面，上半年建成常虎高速和东部快速路，完成莞长、莞惠、莞龙、东深四条主干道改造；加快建设西部干道，改造北王、中麻等公路，开工建设环莞快速路一期、惠常高速谢岗段、莞深高速三期石碣段；积极与省协调，落实沿江高速、博深高速东莞段的建设项目；争取年内完成轨道交通的报批立项；加快客货运站场建设和管理；整治东莞水道42公里，报建万吨级港口泊位7个。此外，要加快建设液化天然气工程，加强燃气安全监管；完成固定电话网号码升8位。

加大农村城市化工作力度。稳妥推进农村股份合作制改革，注意防止任何组织和个人平调、侵占、哄抢、私分集体资产，避免造成集体资产流失和新的分配不公。在确保稳定和群众受益的基础上，积极稳妥地把具备条件的村委会改为居委会，并且切实转变其职能，落实各项社会管理和服务工作。继续推进城镇化三项工作，特别要引导农民建新村退旧村，多村合建节能省地型公寓。

四、克服薄弱环节，提高经济社会管理水平

堵塞经济管理漏洞。强化财政收支管理。推行镇（区）领导班子工商税收目标责任制。抓好重点税源和税种的监控，加强对娱乐、饮食、建材等行业的税收征管，打击偷逃骗税、走私贩私行为，切实做到应收尽收。纳入预算管理的市直行政事业单位要全面实行国库集中收付制度。完成市财政国库集中支付系统与市人大财经工委联网，主动接受其实时监督。强化国有资产管理，加快完善国有资本营运监管制度。加大对行政事业单位资产和政府物业管理力度。继续整顿和规范市场经济秩序。严厉打击制售假冒伪劣产品，保护知识产权。规范建设工程招标投标各方主体行为，建立建设工程保证担保制度。严厉打击建筑领域违法犯罪行为。深化价格改革，整顿收费秩序。加强地方金融机构监管，发展利用资本市场。加强农村集体经济管理，认真开展清产核资，清理和化解不良债务。启动“八镇百村示范工程”。

推进城市管理强化工程。借鉴先进地区经验，构建城市管理长效机制，改进城市管理。推进市政管理市场化，将下水道清淤、公厕管理、交通信号灯和标志牌管理等项目推向市场。加强建筑施工管理，确保文明施工和安全施工，尽量减少对周边生产生活秩序的影响。完善城市交通规划，加强交通设施建设和维护，撤并路桥收费站。落实公交优先发展战略，合理规划公交专用道，开设特快、直快公交。在部分主要路段增设隔离栏，渠化、改造交通繁杂路口，适当增建人行天桥、地下隧道及配套道路设施。市区增设停车位1000多个，设置停车场自动收费系统。继续整顿和规范运输市场秩序。

加强环境与生态保护。编制完成东莞市环境保护、创建生态绿城、大气污染控制、内河涌整治等规划。全面启动36家污水处理厂的建设，建成市区、横沥垃圾焚烧发电厂和全市医疗垃圾集中处理厂，兴建长安、虎门、清溪三项垃圾处理工程。分三个阶段全面整治养殖业污染，全年清理养猪场4443个、家禽场145个。整治一批重点内河涌污染，争取年底基本消除市区运河等主要河段水体“黑臭”现象。整治工业污染，确保9月底前全市电镀、漂染、造纸、制革四大行业236家重点污染企业全面达标排放。启动“国家环保模范城市”创建工作，力争明年达标。初步建成五大森林公园，营造沿海红树林带，保护生态用地，维护生态平衡。推进国营林场和市属水库管理体制改革。

加强人口与计划生育管理。围绕全面完成年度人口和计划生育管理目标，积极推行人口与计划生育工作综合改革，切实完善计划生育利益导向和层级动态管理机制，重点抓好落实生育节育政策、控制出生人口性别比升高、强化流动人口计划生育管理与服务等工作，稳定低生育水平，提高出生人口素质。

五、营造文明法治谅解宽容环境，提高社会和谐程度

提升社会文明水平。以创建“全国文明城市”为龙头，扎实推进群众性精神文明创建活动。进一步加强未成年人思想道德建设。深入开展“爱国、守法、诚信、知礼”现代公民教育和“三现代”教育，大力弘扬东莞精神。

推进基层民主政治建设。进一步健全村（居）民自治机制，推动基层民主政治健康发展。周密安排，统筹兼顾，依法做好村（居）委会换届选举。分批开展村（居）干部培训，提高其理论和业务水平。进一步完善村务公开和民主管理制度。深入开展村民自治示范活动。

大力整顿社会治安。转变“重打轻防”思想，把工作重点、警力配置、经费投入、考核奖惩等转移到预防为主上来。充分发挥人民调解和行政调解的作用，有效化解矛盾。完善出租屋及租住人员管理和服务，深入创建“和谐平安社区”。坚持严打不放松，依法打击严重暴力、“两抢一盗”和黑恶势力等犯罪，坚决清除“黄赌毒”现象。坚持专群结合，把政治强警、素质强警、从严治警和从优待警结合起来，不断提高公安队伍的战斗力；继续增招民警，充实一线警力；充分发动群众参与，推进多种形式的治安联防。试行110、119、122“三台合一”。

认真做好信访工作。积极宣传贯彻国务院新修订的《信访条例》，整合信访资源，建立市信访信息系统，并与省和镇（区）信访信息系统实现互联互通，进一步畅通信访渠道。引导群众依法信访，保护信访人的合法权益，维护信访秩序。进一步完善处理信访突出问题及群体性事件联席会议制度，健全信访督查专员制度、领导包案责任制，抓紧解决信访突出问题。结合“十百千万”干部下基层驻农村工作，各级领导干部要深入排查，积极消除不稳定因素，尽量把问题解决在基层、解决在萌芽状态。

切实加强安全生产。坚持“预防为主”，进一步健全市、镇（区）、村（居）、企业四级安全生产监督管理网络，重点建立镇（区）安全生产监督管理分局。大力推动安全生产工作重心下移，将安全生产责任制度、巡查制度、专项考核制度等全面落实到镇（区）、村（居）和企业，切实提高村（居）和企业注册安全主任持证上岗率。突出对重点企业、重点行业、重点地区的防范和整治，切实消除安全隐患，严防重特大事故发生。有林地的镇（区）要建立专业或半专业森林消防队伍。加强药品和食品质量安全管理，积极防治职业病。

六、发展教科文卫体事业，提高市民综合素质

推进科教兴市。加快建设东莞电子工业研究院，继续举办东莞国际科技合作周。统筹协调，改进科技三项基金和技术改造专项资金的分配使用方法，突出重点，提高实效。办好松山湖留学生创业园，建设松山湖大学科技城，争取再引进2—3所具有较强研发能力的大学，加快把松山湖建成全市的教育、科研和中介服务中心。优化教育资源配置，深化教育改革，创建广东省教育强镇12个。市镇财政增加对农村小学经常性教育经费的投入，进一步明确联办学校教育经费的筹措和管理办法。完善中小学收费管理，加强教育经费使用的监管。巩固发展普及高中教育成果，建成东莞中学松山湖学校、莞城三中新校区，争取东莞中学和市实验中学成为广东省第一批国家级示范性普通高中。鼓励有条件的学校实行初中与高中、职业高中与普通高中分离。推进学校素质教育，增强信息技术和英语教育特色。扩大10所省级以上重点中等职业学校的办学规模，继续推进镇办职业学校联合办学工作。规范发展民办教育，进一步解决好外来员工子女上学问题。积极发展成人教育和高等教育，争取全市高等教育毛入学率达53%以上。推进人才强市战略，积极实施育才工程、聚才工程，引进35名专家，提前两年完成百名专家引进任务。

实施“五大工程”。深入推进学习型城市创建工程、现代市民塑造工程、城市亲和力强化工程、城市文化品牌打造工程和城市现代传媒建设工程，不断提高人的发展能力。加快建设报业大厦、岭南画院等重点文化设施，改造盘活原有文化设施，管好用好新建文化设施。继续深化文化体制改革，撤并组建市文化广电新闻出版局，成立市文化市场综合执法大队，按市场经济体制培育更多文化团体，搞活公益文化事业单位，大力发展文化产业。加强文物博物工作，保护东莞历史文化遗产。实施文艺精品战略，推动高雅艺术与大众文化迅速发展，丰富文化活动。加强文化市场管理，净化文化环境。积极发展哲学社会科学事业。

建设健康城市。完善公益性体育健身设施，发展社区体育，广泛开展群众性体育运动。办好市第六届运动会和全市机关运动会。拓展省运“争金”项目，提高竞技体育实力。科学组织篮球、龙舟等特色体育运动，巩固和发展东莞体育品牌。加紧筹建市体育运动学校新校区。发展体育市场，扩大体育消费，推进体育产业化。加快发展卫生事业，提高医疗卫生水平。落实市公共卫生体系建设规划，加快建设市人民医院新院、中医院、保健院、新涌医院、疾控中心实验综合楼等设施，完善相关工作机制，积极发展社区（村、厂、校）卫生服务站点。联合打击非法行医，规范医疗服务收费和药品购销秩序。加强疾病预防控制，积极防治艾滋病、禽流感等传染病。深入开展创建卫生镇、村（社区）工作，巩固和提高国家卫生城市创建成果。

七、推进民心工程，提高人民生活水平

健全社会保障体系。继续落实“十项民心工程”，认真解决群众看病难、子女读书难等问题。继续完善职工和农（居）民养老、医疗等社会保险制度，积极发展商业保险，建设多层次的社会保险体系。大力发展社会慈善事业和社会公益事业，积极探索敬老院的“公办民营”、“公办民助”等形式，争取两年内全市敬老院达到省级标准，五保户集中供养率达到80%以上。降低欠发达镇村低保金负担比例，市镇财政全额承担原由81个欠发达村负担的农民基本养老保险费和低保金；11个欠发达镇基本医疗救助金市镇分担比例由4∶6调整为6∶4；将基本医疗救助对象的保障范围扩大到五保户、重度残疾人、革命伤残军人、重点优抚对象及其他困难户，将低保家庭在读子女高中阶段的助学金从每年1000元提高到2000元。加大残疾人就业保障金征收力度，切实保护残疾人权益。推进农田水利设施建设，建成东莞大堤达标工程，提高城乡防灾减灾能力。发挥市和镇（区）社会捐助接收站作用，经常性接收社会捐款捐物。

加大扶贫工作力度。落实农村扶贫政策，推进欠发达地区与革命老区的水电路等基础设施建设和联合办学。市财政安排5815万元专项经费，支持全市262个自然村的公路建设，争取年底前村村通水泥公路。增加财政贴息6391万元，继续支持11个欠发达镇发展工业。

扩大就业再就业。加强舆论宣传，改革集体福利分配方式，引导全社会特别是农民转变就业观念。加快实施城乡统一的就业制度，推进农村劳动力转移就业，统筹做好流动就业、涉外就业的管理和服务。强化就业再就业工作目标责任制考核。市财政安排2000万元专项资金，落实再就业技能培训、社保补贴和岗位补贴、小额担保贷款等扶持政策。完善市镇公共就业服务体系，重点加强镇级就业服务平台建设。推广随岗培训、创业培训、订单式培训等有效方式，提高就业成功率。计划全年培训8000人，推荐就业1.8万人。调高企业职工最低工资标准至每月574元，督促企业贯彻执行，畅通投诉渠道，完善法律援助服务，完善工资拖欠预防和监督机制。

坚持为群众办实事。今年要抓好与人民生活密切相关、群众极为关心的10件实事：一是清理违章建筑、乱搭窝棚和养殖业污染，清理无证照经营；二是改善社会治安状况；三是改善供电供水状况；四是改善交通状况；五是初步建成省教育强市；六是提高绿化美化水平；七是建成三项垃圾处理工程；八是建成市农业检测防疫综合大楼；九是加强农民就业服务；十是保护外来员工合法权益。

八、强化政府自身建设，提高政府行政能力

重点推进科学行政。在继续抓好经济调节、市场监管的同时，更加注重社会管理和公共服务，切实投入更多的人力、物力和财力。进一步完善公众参与、专家论证和政府决策相结合的决策机制，保证决策的科学性和正确性。认真编制“十一五”规划。改进和完善政绩考核办法，探索建立政府绩效评估体系和经济社会发展综合评价体系。深化政府机构改革，继续推进电子政务建设和事业单位管理体制改革等工作。加强统计监督。建立健全各种监测、预警和应急机制，提高保障公共安全和处置突发事件的能力。扎实开展保持共产党员先进性教育和“理想、责任、能力、形象”教育，完善公务员选人用人机制和考核机制，加强公务员队伍建设。坚持和完善改进机关作风六项制度，提高政府行政效能。切实贯彻精简、高效、节约的原则，大力改进会风文风。

努力推进民主行政。坚持市长约见市人大代表、会见市政协委员制度，坚决落实市人大及其常委会的决议和决定，及时办理人大议案、建议和政协提案，积极支持市政协履行政治协商、民主监督、参政议政职能，主动听取各民主党派、工商联、无党派人士以及各人民团体的意见和建议。加强内部监督，支持监察、审计部门依法独立履行监督职责。加快建立健全新闻发布制度，设立各级新闻发言人，实行市级新闻发布会和专题新闻发布会制度，进一步接受群众和媒体的监督。

全面推进依法行政。认真贯彻国务院《全面推进依法行政实施纲要》。积极配合市人大及其常委会做好地方立规工作。深入开展行政管理体制、行政决策机制、行政执法机制、行政监督机制和法制宣传教育机制等方面的改革。严格按照法定权限和程序行使权力、履行职责、规范行为，做到有权必有责、用权受监督、侵权须赔偿、违法要追究。加快完善《行政许可法》的配套实施制度，推进行政审批制度改革。总结试点经验，在全市实行主要业务审批“一表通”。规范路上执法，确保实现全市公路基本无“三乱”。探索实行政府法律顾问制度。

深入推进廉洁行政。加快建立健全教育、制度、监督并重的惩治和预防腐败体系。坚决纠正农村征用土地、城镇房屋拆迁、企业重组改制破产等活动中损害群众利益的不正之风，严肃查处利用行政权、人事权、审批权等谋取私利的违纪违法案件。坚持艰苦奋斗、节俭办事，严禁奢侈享乐和铺张浪费。

各位代表！面对新的一年东莞现代化建设的美好蓝图，我们深感责任重大而艰巨；看到全市人民对政府的信任和支持，我们又充满了必胜的信心和力量。让我们高举邓小平理论和“三个代表”重要思想伟大旗帜，齐心协力，求真务实，锐意进取，为圆满完成今年的各项任务、加快建设现代制造业名城而努力奋斗！
